# Supplementary material for: RAMP1 Signaling Mitigates Acute Lung Injury by Distinctively Regulating Alveolar and Monocyte-Derived Macrophages
Source: Int J Mol Sci. 2024 Sep 20;25(18):10107. doi: 10.3390/ijms251810107 (PMC11432488; doi:10.3390/ijms251810107)
Supplement: Supplementary file 1 [file ijms-25-10107-s001.zip › ijms-3223350-supplementary.pdf]

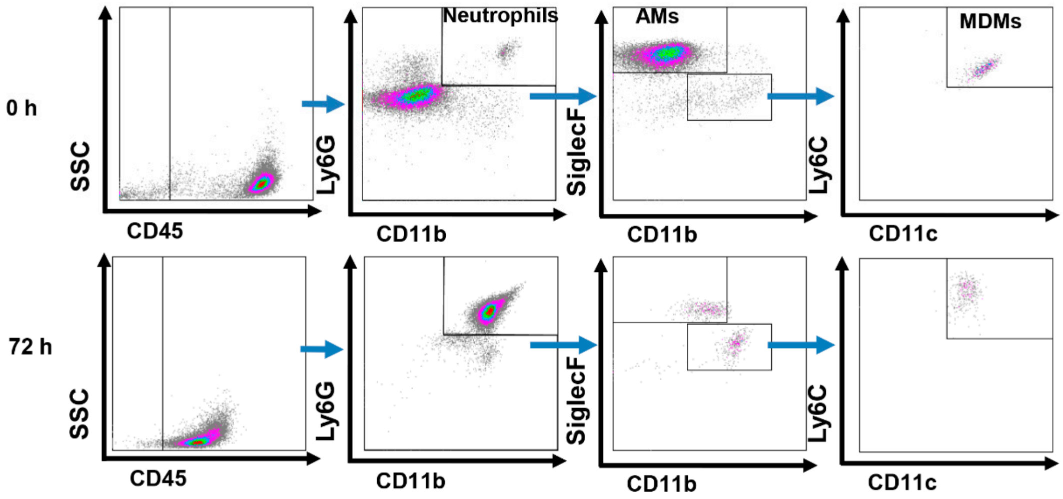

Supplementary Figure S1. Flow cytometry gating strategy used to identify macrophages in LPS-treated mice. After gating of $Ly6G^{high}$ and $CD11b^{high}$ cells (neutrophils), the cells were separated into two subsets based on the expression of SiglecF and CD11b. $Ly6G^{-}$/$SiglecF^{high}$/$CD11b^{low}$ cells were defined as AMs and $Ly6G^{-}$/$SiglecF^{low}$/$CD11b^{high}$ ($Ly6C^{high}$/$CD11c^{high}$) cells were defined as MDMs.

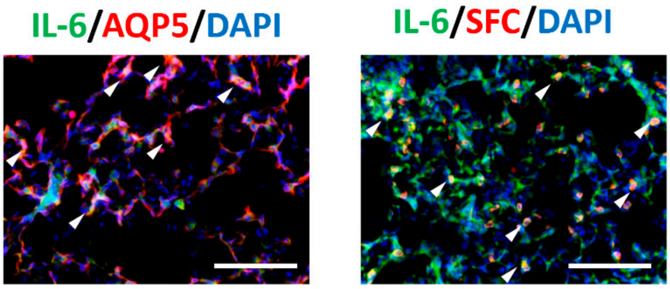

Supplementary Figure S2. Co-localization of IL-6 with pulmonary epithelial cells. Immunofluorescence staining for IL-6 (green) and AQP5 (red) or SFC (red) in lung tissues from WT mice at 72 h after LPS administration. Arrow heads indicate double-stained cells. Cell nuclei were stained with DAPI (blue). Scale bars: 50 μm.

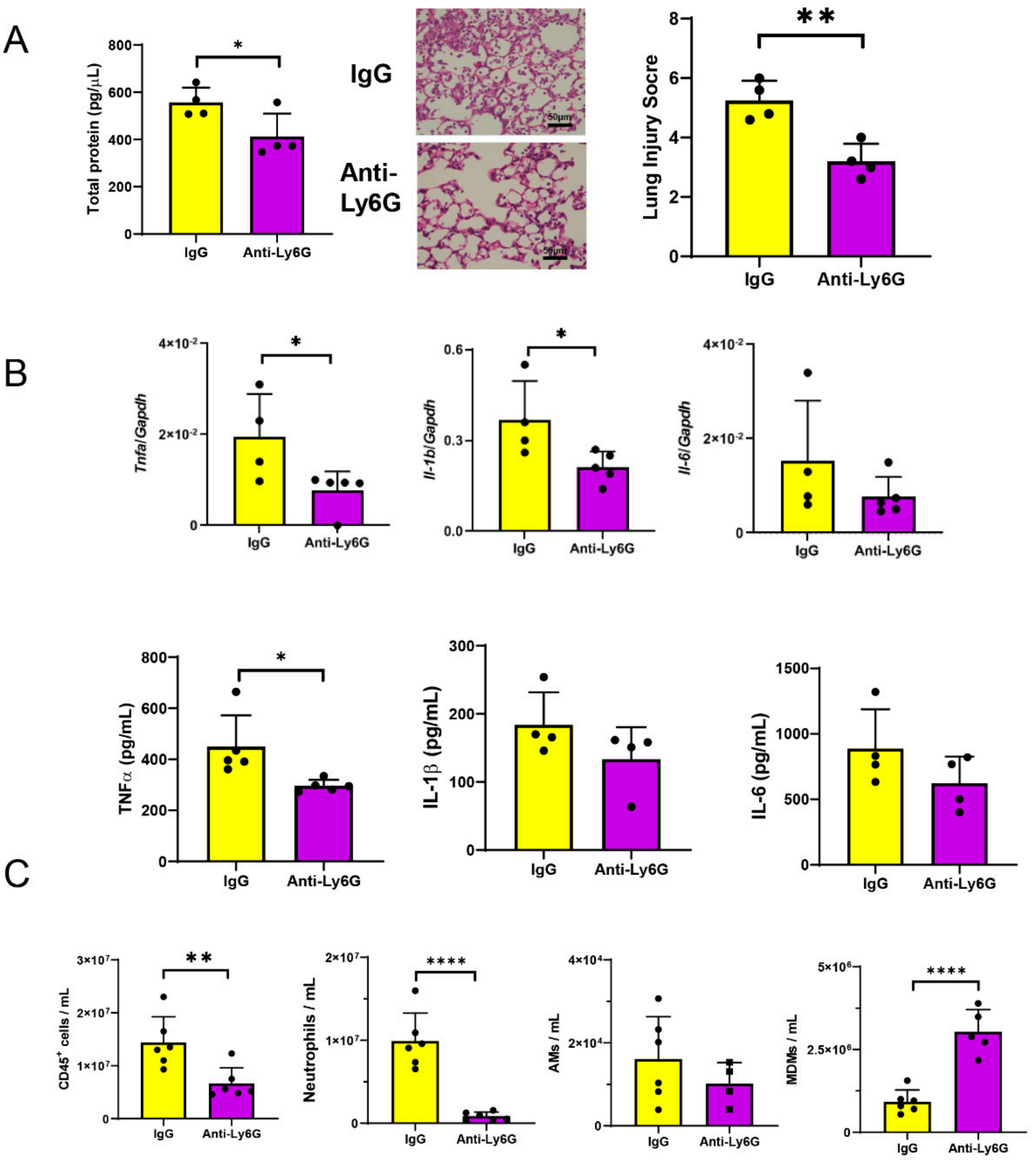

Supplementary Figure S3. Inhibition of neutrophil activity with anti-Ly6G antibody mitigates LPS-induced acute lung injury.

(A) Total protein levels in the BALF and lung injury score at 72 h in WT mice treated with anti-Ly6G antibody or control IgG. Data are expressed as the mean ± SD. * $p < 0.05$, ** $p < 0.01$. Representative images of H&E-stained lung sections at 72 h in WT mice treated with anti-Ly6G antibody or control IgG. Scale bars: 50 μm. (B) Levels of the pro-inflammatory cytokines TNF-α, IL-1β, and IL-6 in lung tissues (upper) and BALF (bottom) at 72 h in WT mice treated with anti-Ly6G antibody or control IgG. Data are expressed as the mean ± SD. * $p < 0.05$. (C) Numbers of $CD45^+$ cells, neutrophils, AMs, and MDMs at 72 h in WT mice treated with anti-Ly6G antibody or control IgG. Data are expressed as the mean ± SD. ** $p < 0.01$, **** $p < 0.0001$.

Supplementary Table S1. Primers used for reverse transcription and quantitative PCR.

Gene	Forward primer sequence (5'-3')	Reverse primer sequence (5'-3')
Cgrp	AGGGCTCTAGTGTTCACTGCTC	AGTTGTCCTTCACCACACCTC
Ramp1	CCATCTCTTCATGGTCACTGC	AGCGTCTTCCCAATAGTCTCC
Tnfa	TCTTCTCATTCCTGCTTGTGG	GATCTGAGTGTGAGGGTCTGG
Il1b	TACATCAGCACCTCACAAGCA	CCAGCCCATACTTTAGGAAGA
Il6	CAAAGCCAGAGTCCTTCAGAG	TAGGAGAGCATTGGAAATTGG
Il10	CGGAAATGATCCAGTTTTACC	TGAGGGTCTTCAGCTTCTCAC
Cxcl2	ATCCAGAGCTTGAGTGTGACG	GCCTTGCCTTTGTTCAGTATC
Ccl2	CGGAACCAAATGAGATCAGAA	TTGTGGAAAAGGTAGTGGATG
Ccr2	TTACCTCAGTTCATCCACGGC	CAAGGCTCACCATCATCGTAG
Gmcsf	CAAGGAACTTCTTGCCAATCCAG	CCAAGATCCACAGGCAAAGCCA
Gapdh	ACATCAAGAAGGTGGTGAAGC	AAGGTGGAAGAGTGGGAGTTG
